# Treatment of Persistent Gastrocutaneous Fistula After Percutaneous Endoscopic Gastrostomy Using Endoscopic Suturing With Argon Plasma Coagulation: A Report of Two Cases

**DOI:** 10.7759/cureus.113653

**Published:** 2026-07-30

**Authors:** Miles J Washington, Morris J Washington, Saptarshi Biswas

**Affiliations:** 1 Medical Education, University of South Carolina School of Medicine Columbia, Columbia, USA; 2 Gastroenterology, McLeod Seacoast/Loris Medical Center, Little River, USA; 3 Trauma and Acute Care Surgery, Grand Strand Regional Medical Center, Myrtle Beach, USA

**Keywords:** argon plasma coagulation (apc), endoscopic approach, endoscopic clip, endoscopic suturing, gastrocutaneous fistula, overstitch device, percutaneous endoscopic gastrostomy (peg) feeding, polypropylene suture

## Abstract

Persistent gastrocutaneous fistula (PGCF) is an uncommon but often challenging complication following removal of a percutaneous endoscopic gastrostomy (PEG) tube. Prolonged PEG tube requirement leads to epithelialization of the PEG tube tract, resulting in a PGCF when the tube is removed. Several management options have been described for this complication, many of which demonstrate variable success rates. However, no definitive treatment for PGCF has been established. We report two adult male patients, aged 63 and 30 years, who developed PGCF after PEG tube removal following prolonged PEG placement. The first patient had persistent low-volume drainage for approximately one year after removal of the PEG tube, despite an attempt at external purse-string suture closure with simultaneous endoscopic clip placement. The second patient developed persistent high-volume drainage with peristomal skin breakdown despite local wound care and ostomy appliance placement for 3 months after removal of the PEG tube. Both patients underwent argon plasma coagulation of the fistula tract followed by endoscopic suture closure using the Boston Scientific OverStitch device (Boston Scientific Corporation, Marlborough, USA). In both cases, fistula drainage resolved within one week, with no documented recurrence during available follow-up. These cases are reported to highlight the technical feasibility of combined argon plasma coagulation and endoscopic suturing for selected chronic post-PEG PGCFs and to add to the limited published experience with this approach

## Introduction

Persistent gastrocutaneous fistula (PGCF) is an uncommon but often challenging complication following removal of a percutaneous endoscopic gastrostomy (PEG) tube, occurring in approximately 5-6% of adult patients [1-3]. The incidence of PGCF is directly related to the duration of PEG tube placement. Longer PEG tube dwell times are associated with epithelialization of the fistulous tract, resulting in failure of spontaneous closure after tube removal [4].

Limited external surgical closure of the fistulous tract, typically performed under local anesthesia with purse-string suture repair, is associated with a high recurrence rate [5,6]. More extensive surgical approaches involving laparotomy/laparoscopy with takedown of the fistulous tract and stapled or sutured gastric closure have demonstrated high success rates, but they are major surgical procedures associated with significant operative stress and recovery in an already frail patient population. Endoscopic techniques using standard through-the-scope clips (TTSCs) and over-the-scope 'bear claw' clips (OTSCs) have also demonstrated relatively high failure rates in the treatment of chronic fistulas, with failure rates of 40-60% [7-10].

We report two cases of PGCF following PEG tube removal that were successfully treated with a combination of endoscopic suture closure (ESC) using the OverStitch device (Boston Scientific, Marlborough, USA) and argon plasma coagulation (APC). A review of the current literature identified two additional reported cases of PGCF after PEG tube removal that were successfully treated using this technique [1,2].

Because published experience with combined APC and ESC for post-PEG PGCF remains limited, additional case documentation may help clarify patient selection, technical considerations, and short- to intermediate-term outcomes. We present two medically complex patients with chronic PGCF after PEG removal who were treated with APC-assisted OverStitch closure. These cases are intended as a focused case series and narrative discussion of a technically reproducible endoscopic approach, rather than evidence of comparative efficacy.

## Case presentation

This retrospective case series describes two patients with persistent gastrocutaneous fistula after PEG tube removal who underwent endoscopic closure using APC followed by OverStitch endoscopic suturing at our institution. Cases were identified retrospectively from clinical experience and chart review. Both patients had persistent fistula drainage after PEG removal despite initial conservative or procedural management. Available clinical records were reviewed for patient characteristics, PEG dwell time, prior management, procedural technique, clinical resolution of drainage, recurrence, and documented follow-up. Because this was a retrospective two-patient case series, there was no pre-specified protocol, comparator group, or standardized follow-up schedule.

Case 1

The patient was a 63-year-old male who underwent repair of a Type A aortic dissection. His postoperative course was complicated by respiratory failure requiring percutaneous tracheostomy and PEG tube placement. Recovery was further complicated by renal failure requiring dialysis, sternal wound infection, and anoxic encephalopathy.

Despite these complications, the patient recovered and was ultimately decannulated and subsequently underwent PEG tube removal. The PEG tube had remained in place for 17 months. The presenting complaint was persistent drainage from the prior PEG site after eating, with surrounding skin irritation. Clinical assessment was consistent with PGCF, given the history of PEG removal, persistent drainage from the gastrostomy site with meals, and endoscopic visualization of the fistula opening. Alternative considerations included superficial wound drainage, localized infection, or incomplete cutaneous healing; however, persistence of drainage and identification of the gastric opening supported the diagnosis of PGCF.

After one year of intermittent but persistent fistula drainage, an initial attempt at closure was performed. The external fistulous tract was closed under local anesthesia using a purse-string 3-0 polypropylene suture. Concurrent upper endoscopy was performed, and TTSCs were placed across the internal fistula opening. This intervention failed to reduce fistula output.

**Figure 1:**
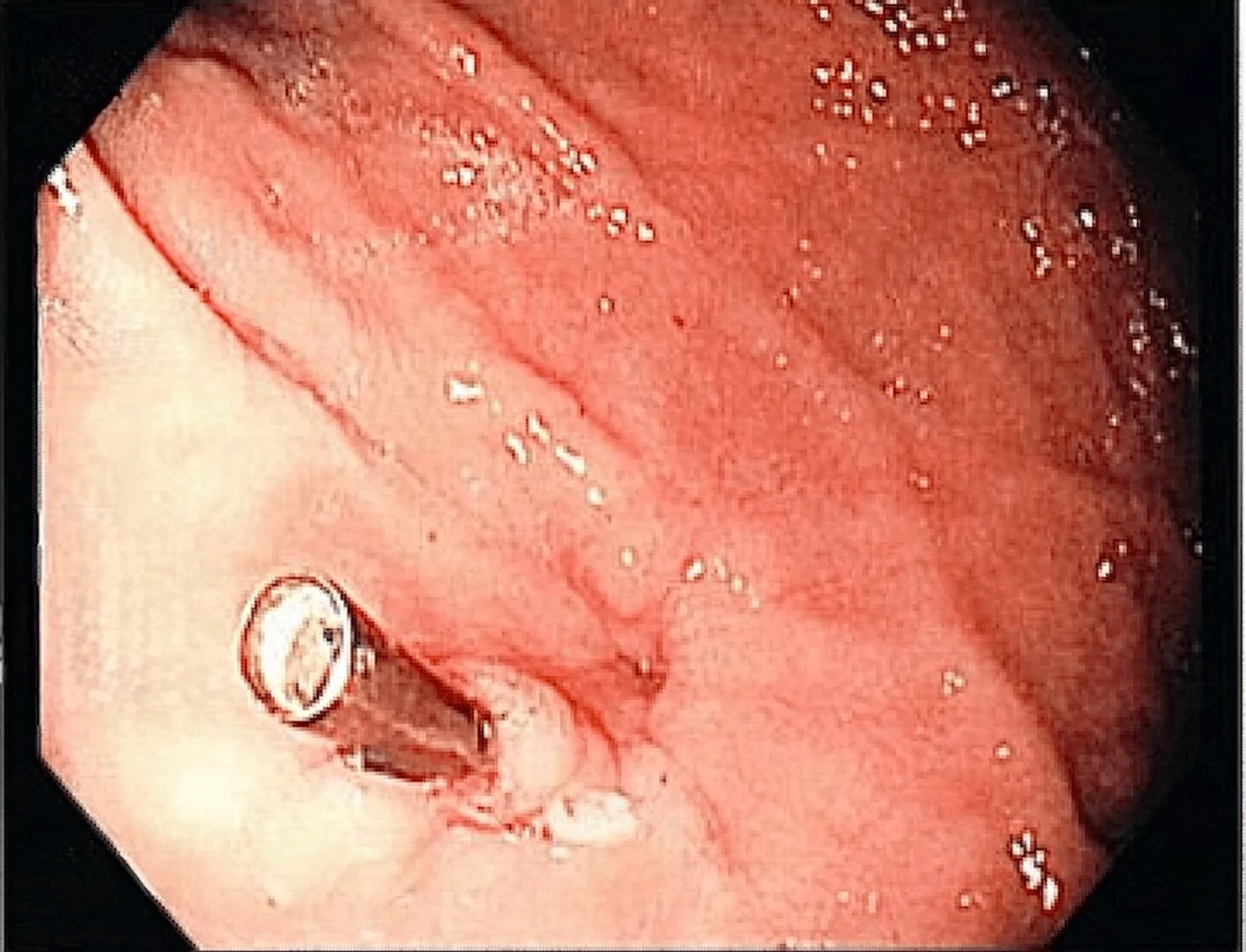
Fistula opening with endoclip from a prior failed closure attempt

**Figure 2:**
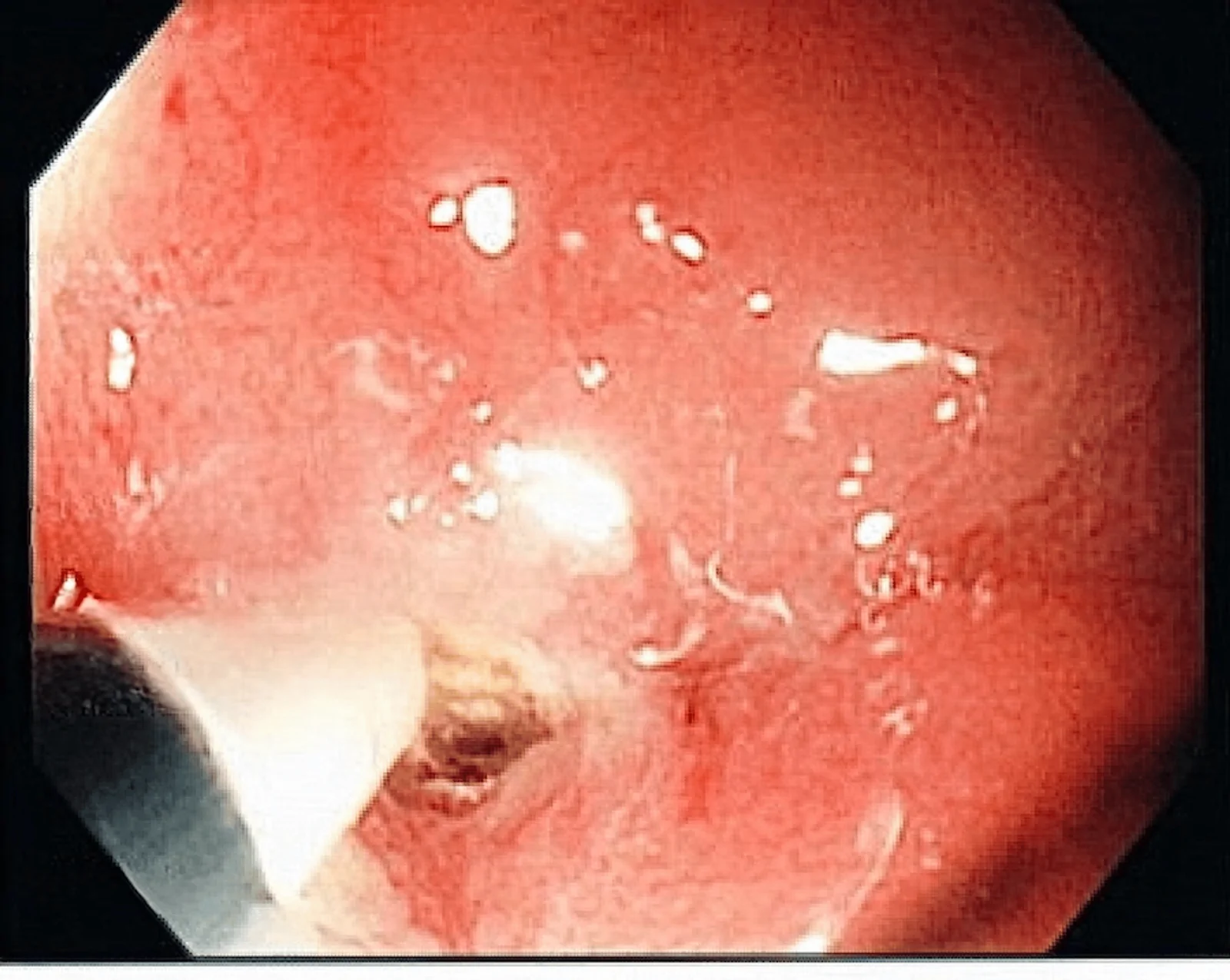
Argon plasma coagulation (APC) of fistulous tract prior to suture closure

**Figure 3:**
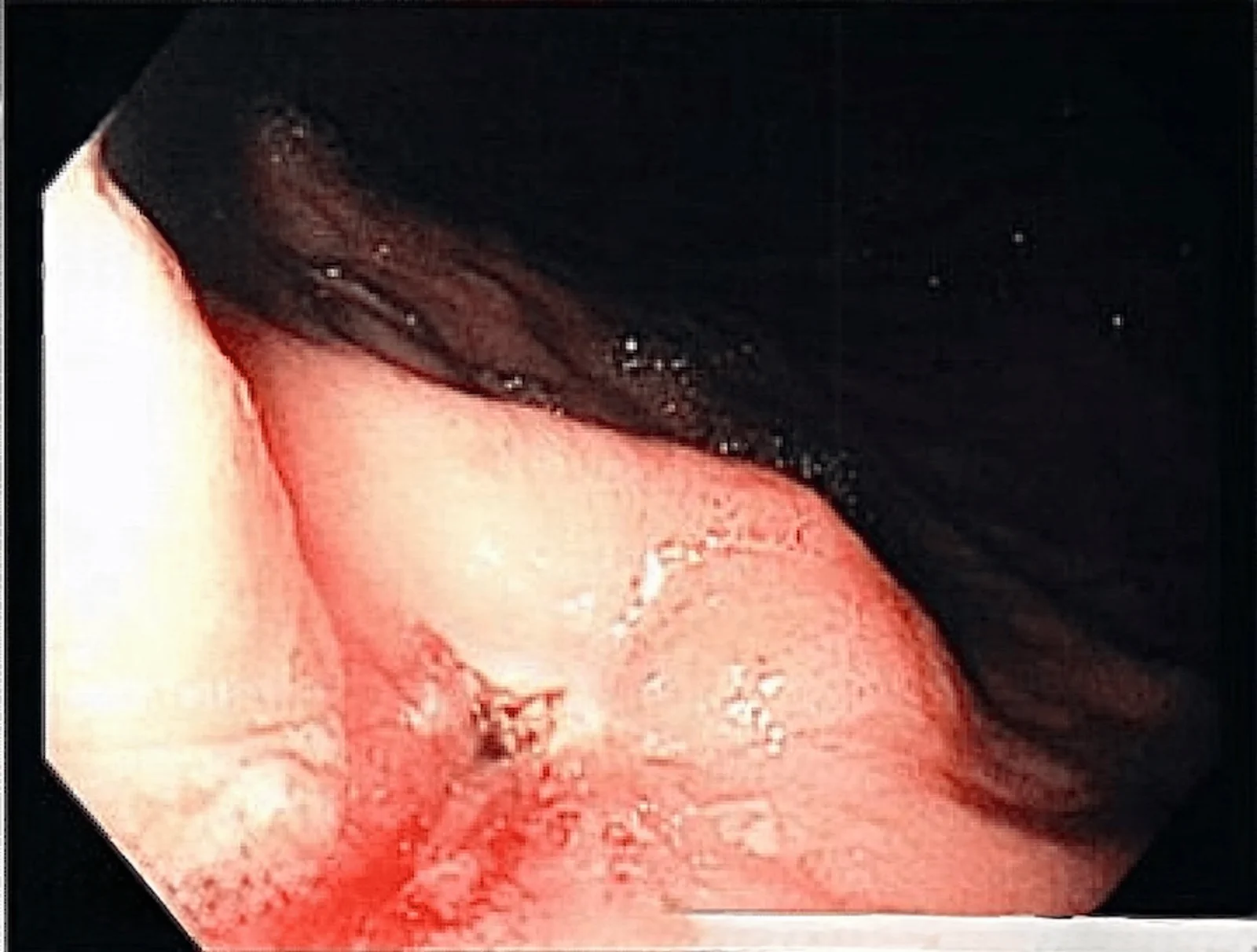
Fully coagulated fistula tract in preparation for suture closure

**Figure 4:**
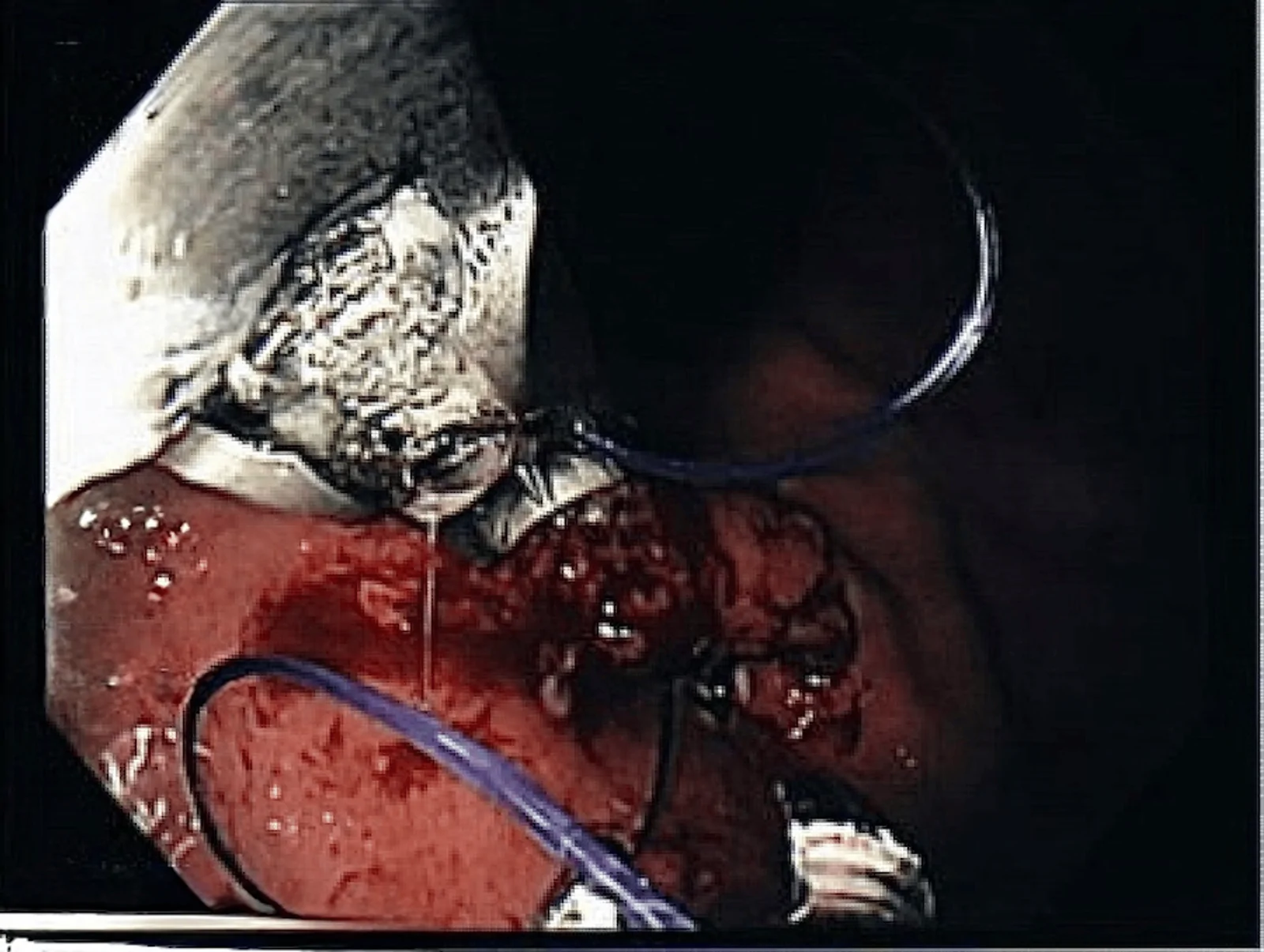
Suture closure of fistula tract using the OverStitch device and 2-0 polypropylene suture OverStitch device by Boston Scientific,* *Marlborough, USA

**Figure 5:**
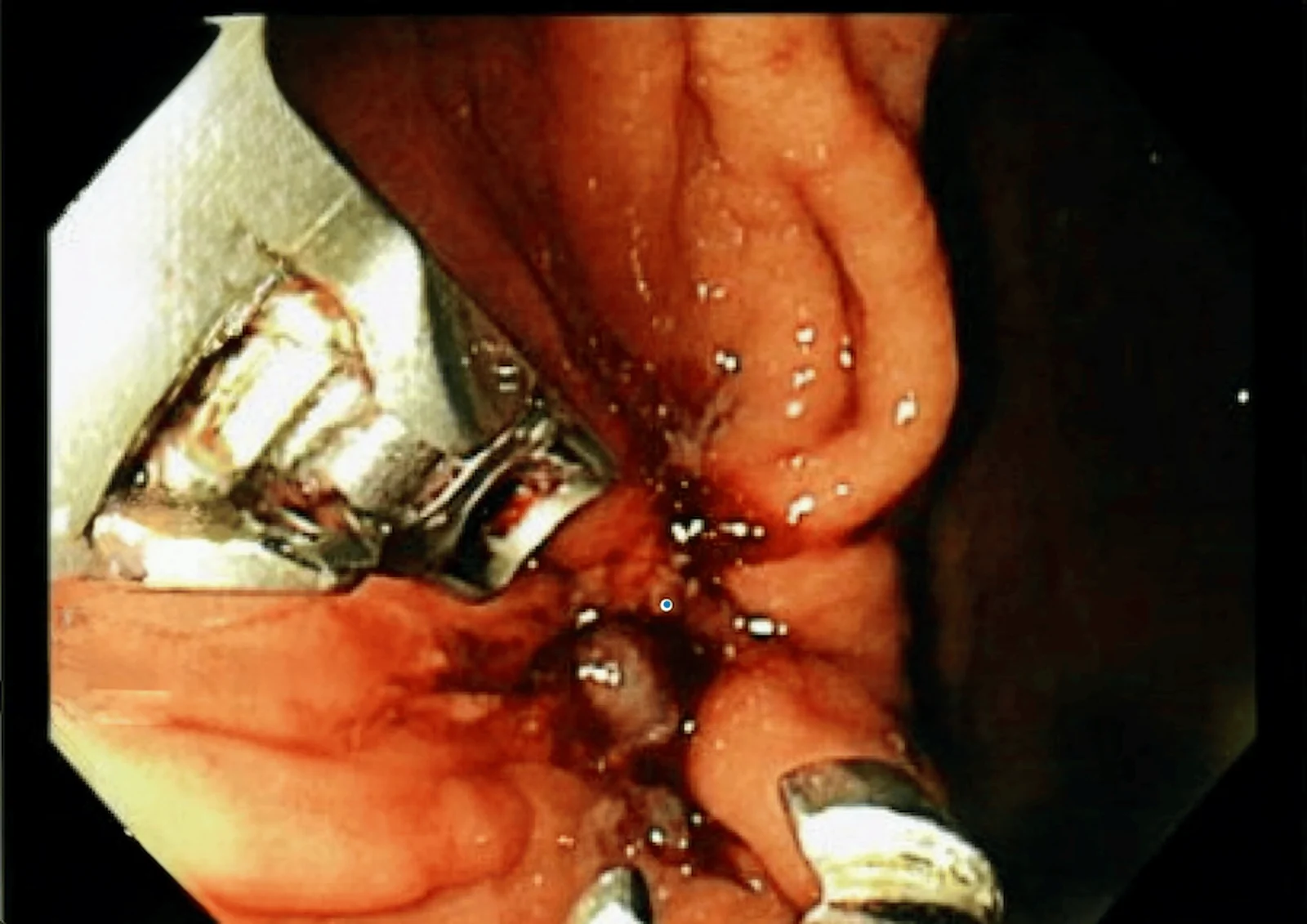
Final appearance of closed fistula

One month after the initial attempt, the patient underwent ESC combined with APC as an outpatient (Figures 1-5). Within one week, fistula drainage had completely resolved, with no recurrence observed during 3 years of follow-up. There was no procedure-related bleeding, perforation, infection, hospitalization, or need for repeat intervention after the procedure.

Case 2

The patient was a 30-year-old man with morbid obesity (BMI 61.8) and obstructive sleep apnea who was admitted with sepsis secondary to an abscess of the right lower extremity with cellulitis. During hospitalization, he developed pneumonia complicated by respiratory failure requiring endotracheal intubation. He subsequently underwent tracheostomy and PEG tube placement for prolonged ventilatory and nutritional support. His clinical course was further complicated by a sacral decubitus ulcer requiring frequent debridement and vacuum-assisted wound therapy.

After a prolonged hospitalization, the patient was successfully weaned from mechanical ventilation and ultimately resumed oral intake. The PEG tube was removed eight months after placement. Following PEG removal, he developed a high-output PGCF associated with significant peristomal skin breakdown. Despite local wound care and placement of an ostomy appliance over the fistula site, he continued to have persistent high-output drainage and progressive skin irritation. Three months after PEG removal, the fistula remained patent with ongoing skin breakdown and irritation.

The clinical diagnosis of PGCF was based on persistent high-output gastric drainage from the PEG tube site after PEG removal and endoscopic identification of the fistula opening.

**Figure 6:**
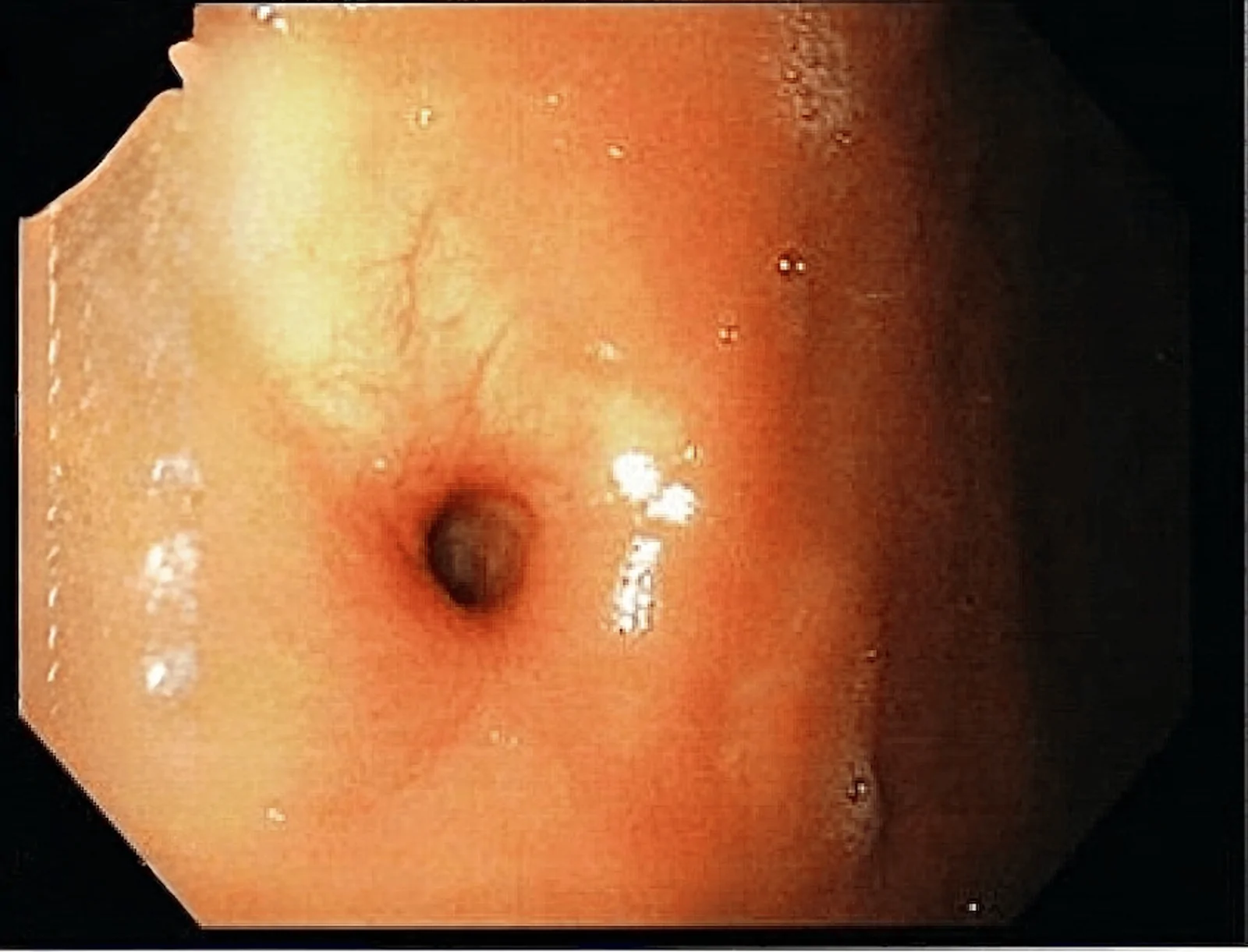
Fistula opening

**Figure 7:**
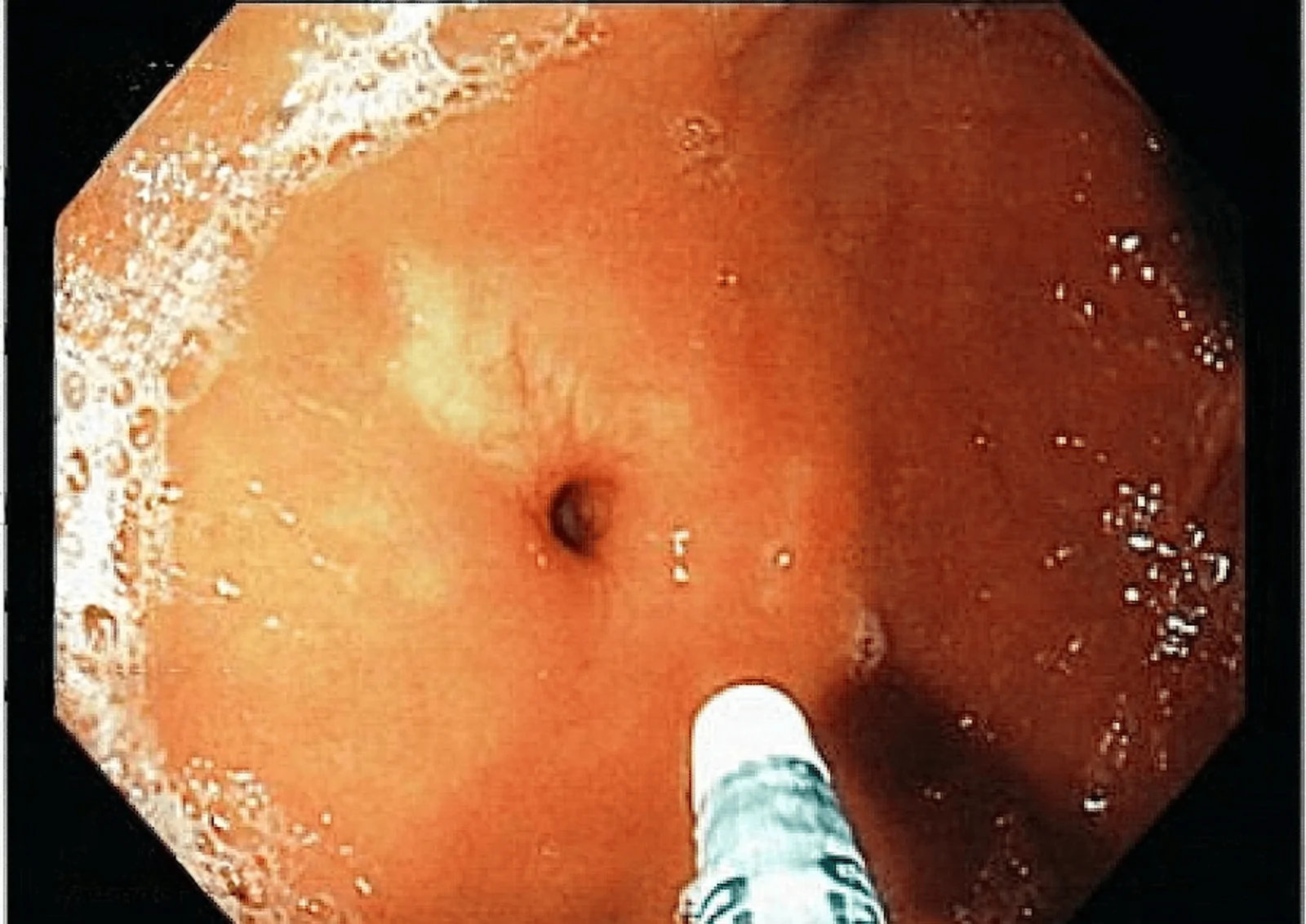
Argon plasma coagulator (APC) probe introduced through endoscope

**Figure 8:**
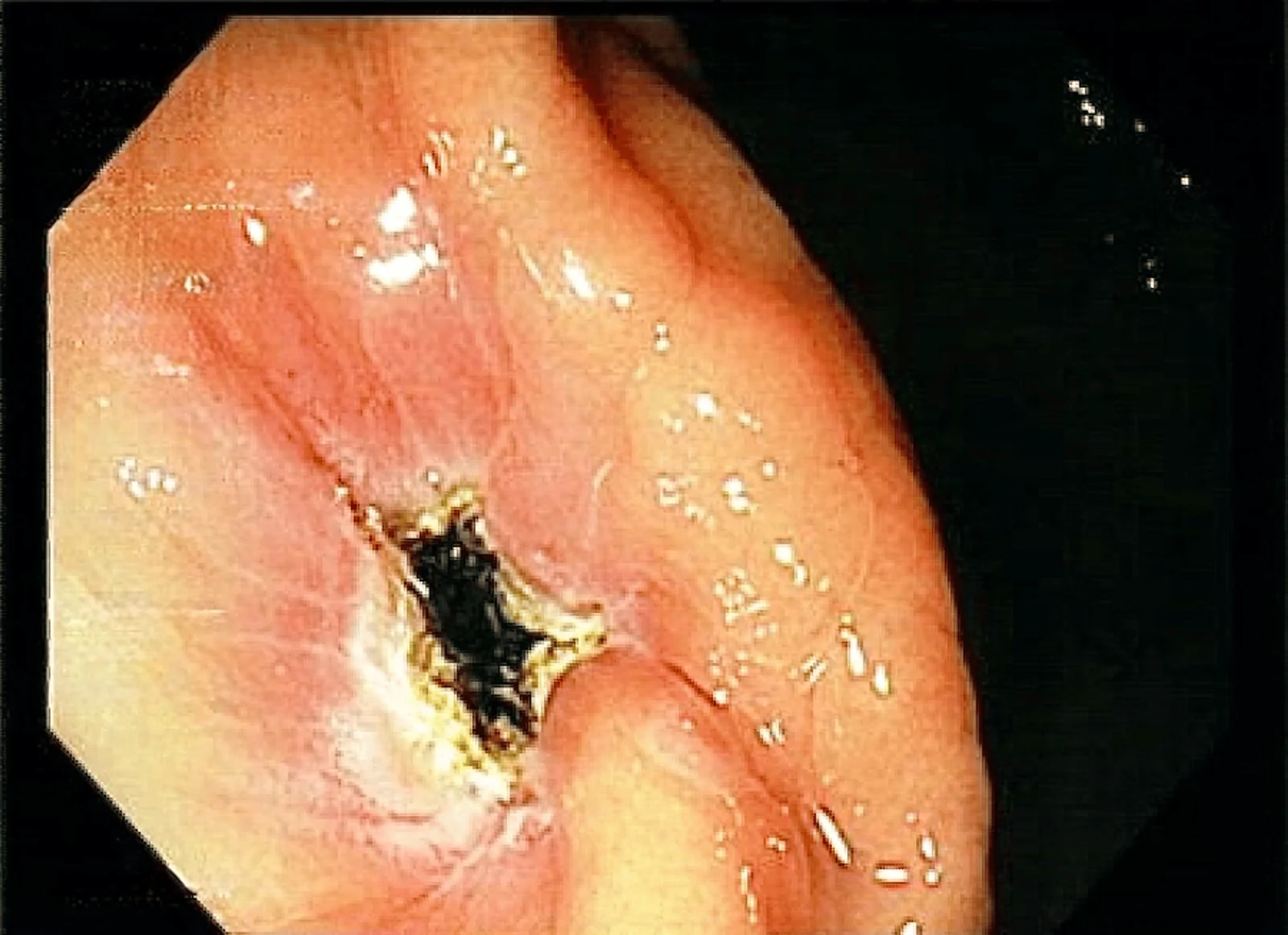
Fully coagulated fistula tract in preparation for suture closure

**Figure 9:**
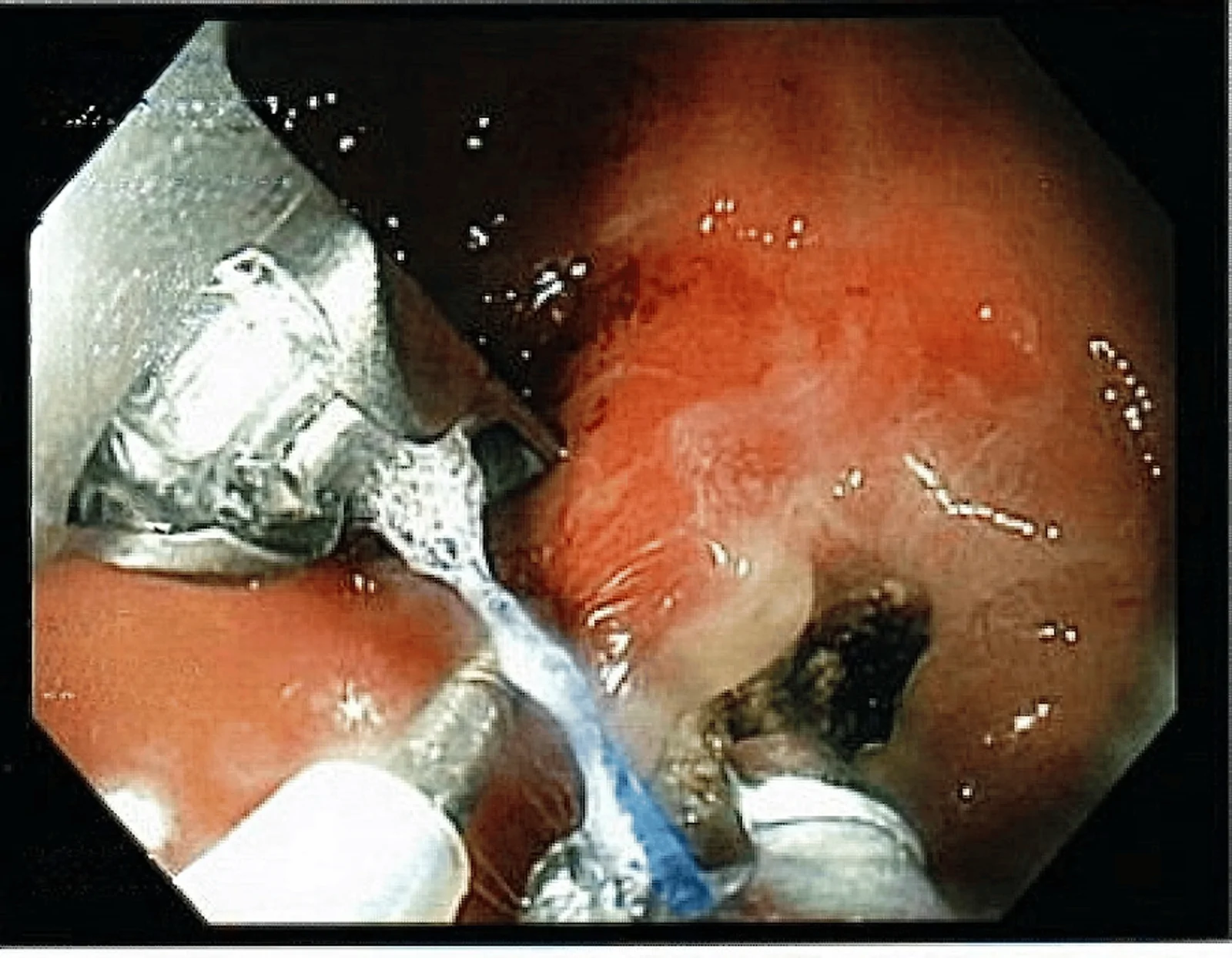
Suture closure of fistula tract using the OverStitch device and 2-0 polypropylene suture OverStitch by Boston Scientific Corporation, Marlborough, USA.

**Figure 10:**
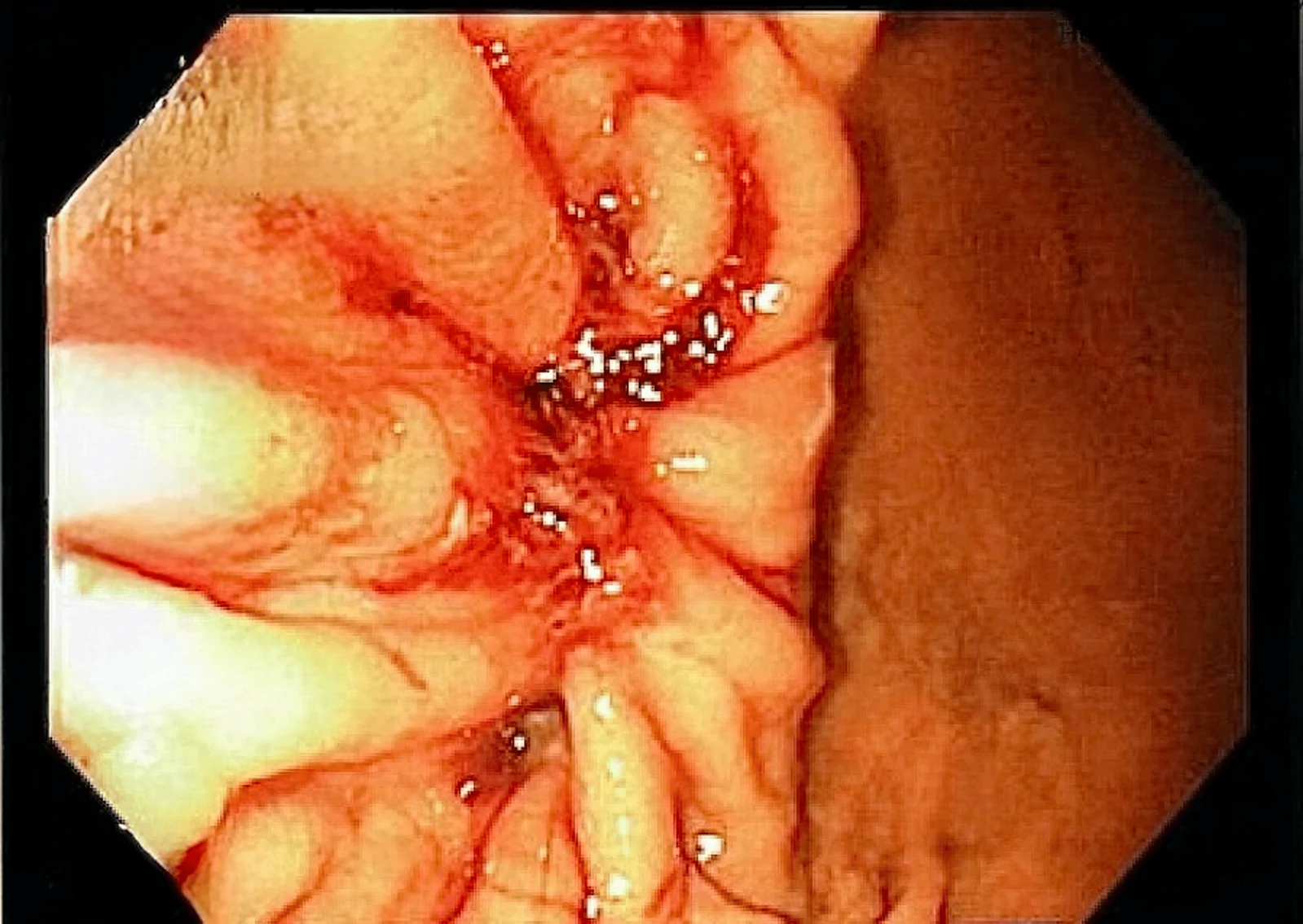
Final appearance of the closed fistula

The patient subsequently underwent ESC combined with APC as an outpatient (Figures 6-10). Complete resolution of fistula drainage was achieved within one week of the procedure, and no recurrence was observed during one year of follow-up. There was no procedure-related bleeding, perforation, infection, recurrence, hospitalization, or need for repeat intervention after the procedure.

Technique

**Figure 11:**
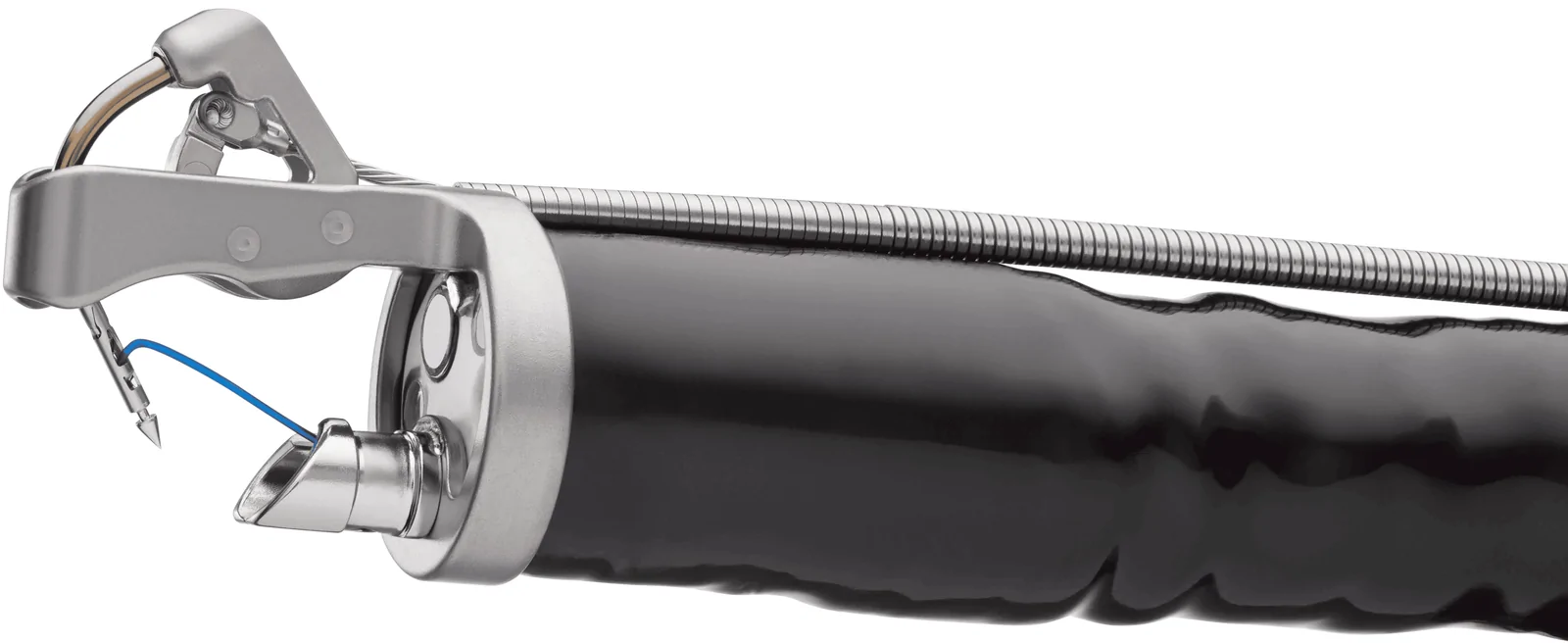
Boston Scientific OverStitch device Source: Image provided courtesy of Boston Scientific. © 2026 Boston Scientific Corporation and its affiliates. All rights reserved.

**Figure 12:**
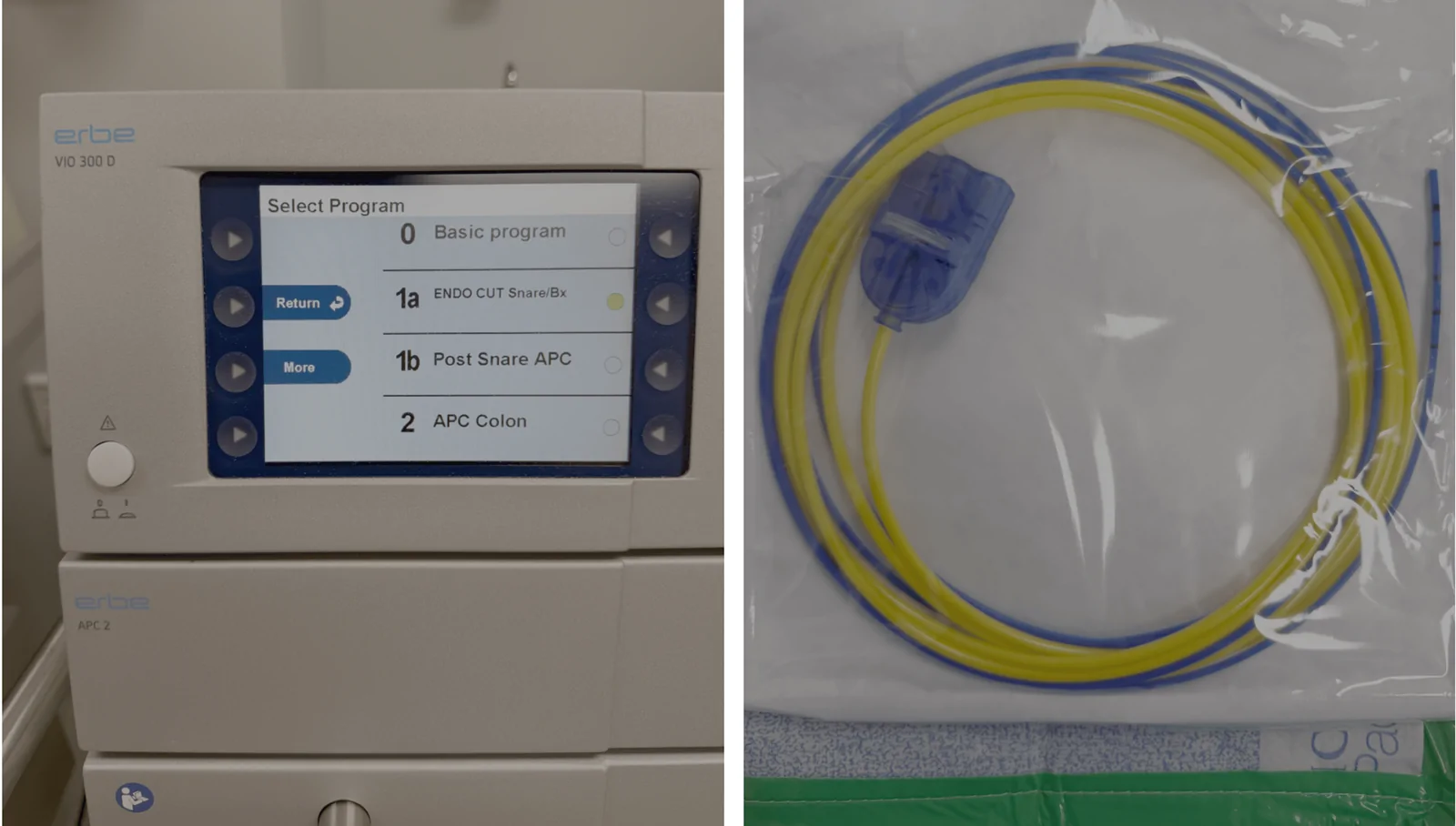
Argon plasma coagulator (APC) probe Argon plasma coagulator probe by ERBE USA Inc., Marietta, USA. Source: Photographs by the authors.

The procedures were performed under general endotracheal anesthesia using propofol anesthesia. A double-channel therapeutic gastroscope (Olympus GIF-2TH180; Olympus Corporation, Tokyo, Japan), the OverStitch endoscopic suturing device (Boston Scientific) (Figure 11), and an argon plasma coagulation (APC) system (Erbe Elektromedizin GmbH, Tübingen, Germany) (Figure 12) were utilized. Patients were positioned in the left lateral decubitus position, as is standard for upper endoscopy.

The gastroscope was advanced through the esophagus into the stomach, and the fistula opening and tract were examined. The mucosa surrounding the fistula opening and tract was ablated using APC at a power setting of 50 W, a flow rate of 1 L/min, and 1-second pulses until the tract mucosa was adequately coagulated.

Following APC treatment, the fistula was closed using the OverStitch device with 2-0 polypropylene suture. Full-thickness bites were placed across the fistula opening in a figure-of-eight pattern to achieve closure. An additional full-thickness reinforcing simple stitch was then placed. Adequate closure was confirmed by cessation of CO₂ gas leakage from the external cutaneous fistula opening. This approach was adapted from previously described endoscopic suturing techniques for gastrointestinal fistula closure, including reports in which mucosal ablation or de-epithelialization was performed before mechanical closure [1-3].

## Discussion

Patients with prolonged PEG tube placement (>6 months), particularly those with morbid obesity, chronic infection, or malnutrition, are at increased risk for developing PGCFs [5,6]. Prolonged indwelling time promotes epithelialization of the fistulous tract, resulting in a stable channel between the stomach and abdominal wall that is resistant to spontaneous closure. Continuous exposure to gastric contents and ongoing local inflammation may further impair healing, rendering conservative measures-such as acid suppression and local wound care-largely ineffective in many chronic cases.

Several surgical techniques have been described for the management of post-PEG PGCFs. These include limited external suture closure of the fistulous tract, laparoscopic stapled transection, and open surgical repair [5,6]. Limited external surgical closure has been associated with relatively high recurrence rates [5,6]. While laparoscopic and open surgical approaches are generally effective, they may be poorly tolerated in medically complex patients with significant comorbidities [5,6]. Alternative approaches, including fibrin sealants, endoscopic band ligation, and percutaneous techniques, have also been described for fistula closure with varying levels of success [11-13].

**Figure 13:**
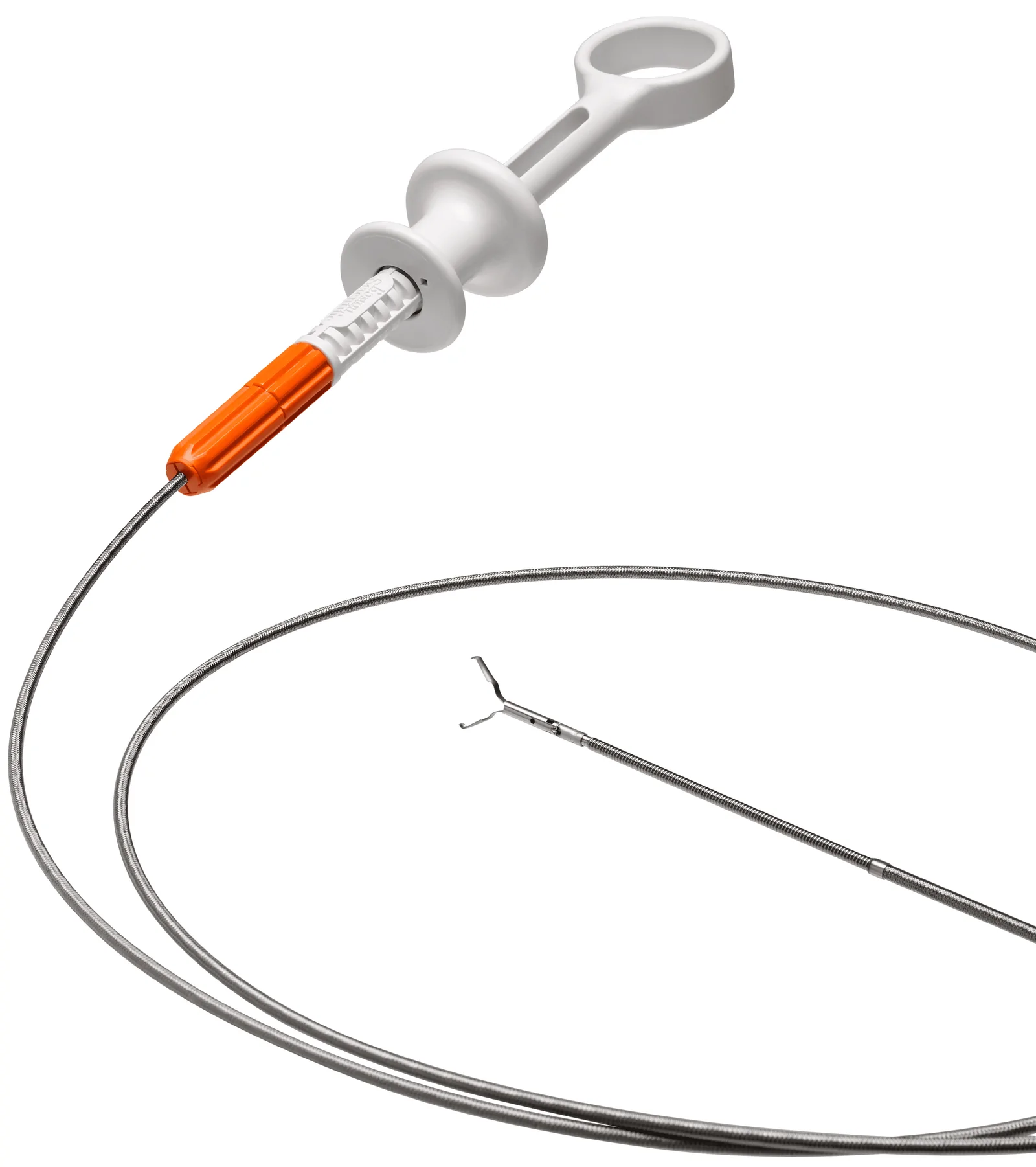
Boston Scientific Resolution Through-the-Scope Clip (TTSC) Source: Image provided courtesy of Boston Scientific. © 2026 Boston Scientific Corporation and its affiliates. All rights reserved.

**Figure 14:**
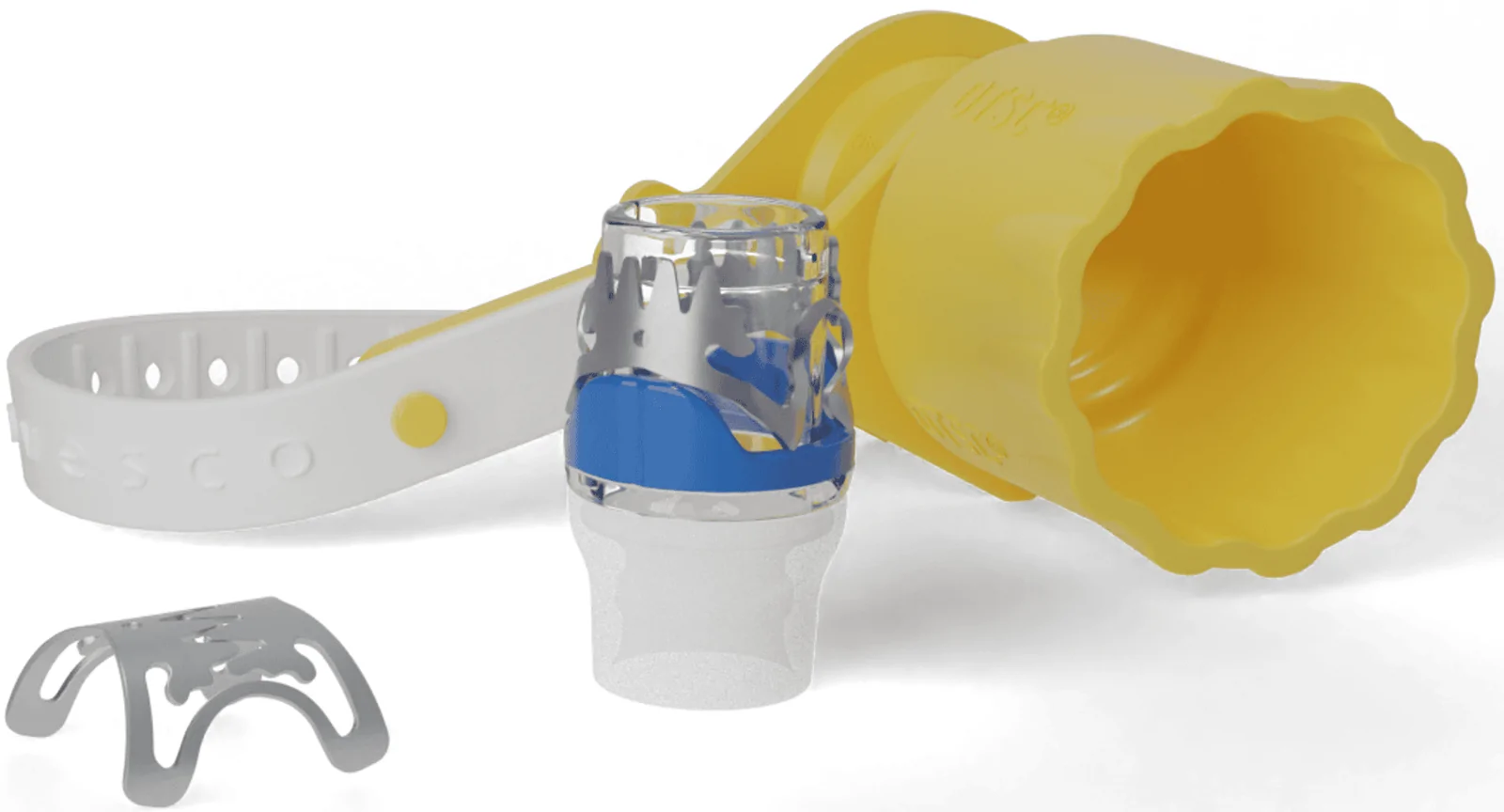
Ovesco Over-the-Scope Clip (OTSC) Source: Photo courtesy of Ovesco Endoscopy AG*.*

Endoscopic management is increasingly utilized for the treatment of post-PEG PGCFs. TTSCs and OTSCs (Figures 13, 14) have demonstrated success in selected patients, particularly those with smaller or more acute fistulas; however, efficacy appears to decline in chronic, epithelialized, or fibrotic tracts [7-10]. TTSCs primarily approximate mucosal tissue, and even OTSC systems may fail to achieve durable closure when significant fibrosis is present. Tissue sealants, including fibrin glue, and fistula plugs have also been employed, although reported outcomes have been variable [11-13].

Endoscopic suture closure (ESC) using the Boston Scientific OverStitch device has emerged as a promising alternative for the management of persistent PGCF. Although published data remain limited, prior reports suggest encouraging outcomes. Armengol-Miró et al. described a case in which argon plasma coagulation (APC) was used to de-epithelialize the tract prior to ESC, resulting in complete closure with no recurrence at 6-month follow-up [1]. Kantsevoy and Thuluvath reported successful closure of a PGCF using ESC after failed clip-based therapy, with durable healing at 3 months [2]. A 2021 conference abstract similarly demonstrated successful closure following combined APC and ESC therapy [3]. These reports demonstrate consistent short-term success; however, long-term data and standardized technical approaches remain limited, underscoring the need for further case documentation, including the present report.

The present cases are consistent with prior reports describing successful PGCF closure using ESC, with or without adjunctive APC. Similar to the previous reports, both of our patients had chronic fistulas after prolonged PEG placement and achieved clinical resolution shortly after endoscopic therapy. Together, these reports suggest that APC-assisted ESC may be technically feasible in selected chronic PGCFs, but the evidence remains limited to small case reports and retrospective observations.

To better compare the present cases with the limited published experience, previously reported cases using ESC with or without APC for post-PEG PGCF are summarized in Table 1.

**Table 1:** Published cases using endoscopic suturing with or without APC for post-PEG persistent gastrocutaneous fistula. OverStitch device by Boston Scientific Corporation, Marlborough, USA. APC: argon plasma coagulation; PEG: percutaneous endoscopic gastrostomy; PGCF: Persistent gastrocutaneous fistula; TTSC: through-the-scope clip

Study	Technique	Clinical context	Outcome/follow-up	Comparison with present cases
Armengol-Miró et al. [1]	APC followed by endoscopic suturing	Chronic gastrocutaneous fistula in an immunocompromised patient	Complete closure; no recurrence at 6 months	Similar use of APC-assisted suturing; present cases add longer follow-up in one patient
Kantsevoy and Thuluvath [2]	Endoscopic suturing after failed clip-based therapy	Chronic refractory gastrocutaneous fistula	Durable closure at 3 months	Similar to Case 1, which also followed failed closure/clip therapy
2021 conference abstract [3]	APC followed by endoscopic suturing	Persistent gastrocutaneous fistula after PEG removal	Successful closure reported	Similar combined approach, but limited details due to abstract format
Present Case 1	APC followed by OverStitch closure	Low-volume chronic PGCF after 17-month PEG dwell time; failed external suture closure and TTSCs	Drainage resolved within 1 week; no recurrence at 3 years	Adds longer documented follow-up after failed prior therapy
Present Case 2	APC followed by OverStitch closure	Clinically high-volume PGCF after 8-month PEG dwell time with peristomal skin breakdown	Drainage resolved within 1 week; no recurrence at 1 year	Adds experience in a patient with substantial skin breakdown and clinically higher-volume drainage

Overall, the present cases are consistent with prior reports showing clinical resolution after ESC-based closure of post-PEG PGCF. Similarities include chronicity of the fistula, suspected epithelialization, and use of mechanical closure with or without adjunctive mucosal ablation.

The combination of ESC with APC directly addresses several key limitations of chronic fistulas. APC facilitates de-epithelialization of the tract and may promote granulation tissue formation, while ESC provides full-thickness mechanical closure capable of addressing larger, chronic, or fibrotic defects. In the limited available literature, only a small number of published case reports and one meeting abstract describe ESC for post-PEG PGCF closure, all of which reported successful outcomes [1-3]. Our two additional cases further support this approach and suggest that ESC combined with APC may represent a durable, minimally invasive alternative to clip-based endoscopic techniques for chronic post-PEG PGCFs [1,2,7-10].

Limitations

This report has several limitations. It is a retrospective two-patient case series without a comparator group, randomization, or pre-specified treatment protocol. No conclusions can be drawn regarding comparative efficacy versus conservative management, TTSCs, OTSCs, fibrin sealants, APC alone, ESC alone, or surgical repair. Fistula output was described clinically as low or high output, but objective daily output measurements were not available. Clinical success was assessed by documented cessation of external drainage, skin healing, lack of recurrent drainage, and absence of repeat intervention during available follow-up; routine contrast studies, repeat endoscopic confirmation, histologic confirmation of epithelialization, and standardized patient-reported outcome measures were not performed. Follow-up was based on available clinical documentation rather than a standardized surveillance protocol. Finally, this technique requires specialized endoscopic equipment, operator experience, and anesthesia support, which may limit generalizability. Larger prospective studies are needed to better define patient selection, durability, safety, and comparative effectiveness.

## Conclusions

In this two-patient retrospective case series, APC-assisted endoscopic suture closure using the OverStitch device was technically feasible and was followed by clinical resolution of chronic post-PEG PGCF without documented recurrence during available follow-up. These cases suggest that combined APC and ESC may be a reasonable minimally invasive option in selected patients with persistent, suspected epithelialized fistula tracts, particularly when conservative or clip-based approaches are unsuccessful or unlikely to provide durable closure. Because evidence remains limited to small case reports and case series, larger prospective studies are needed before comparative efficacy or standardized treatment recommendations can be established.
